# Wastewater COD characterization: RBCOD and SBCOD characterization analysis methods

**DOI:** 10.1038/s41598-020-80700-8

**Published:** 2021-01-12

**Authors:** Jingbing Zhang, Yuting Shao, Guohua Liu, Lu Qi, Hongchen Wang, Xianglong Xu, Shuai Liu

**Affiliations:** Research Center for Low Carbon Technology of Water Environment, School of Environment and Natural Resource, Renmin University of China, Beijing, 100872 China

**Keywords:** Biochemistry, Environmental sciences

## Abstract

Wastewater characterization is the basis for process design and operation optimization of wastewater treatment plants (WWTPs). In this work, a comprehensive study of the respirometry method has been performed to evaluate the biodegradable organic matters of wastewater. First, the optimal initial substrate to biomass ratio (S_0_/X_0_) was confirmed. Second, under the optimal S_0_/X_0_, the degradation curves of wastewater carbon oxygen demand (COD) components rapidly biodegradable COD (RBCOD) and slowly biodegradable COD (SBCOD) were obtained. Third, the Mann–Kendall test was performed to confirm the time point (t_2_) when endogenous respiration levels were reached, and the hydrolysis model was used to determine the time point (t_1_) of the SBCOD degradation stage. Considering the results, an adequate wastewater COD characterization method for RBCOD and SBCOD has been proposed. This study provides strong support to carry out effective and feasible process design, process diagnosis and optimization capability, can help achieve refined and stable operational management of WWTPs.

## Introduction

With the increase in urbanization and the rapid development of urban China, the amount of wastewater discharge continues to increase, resulting in deterioration of the water environment and serious pollution of water resources^1^. The wastewater treatment facilities in China have continued to develop at a high speed in the past decade^2^. In addition, increasingly stringent wastewater treatment discharge standards have been implemented to regulate nutrient removal^3,4^, that put forward stricter requirements for the refined design and operation of wastewater treatment plants (WWTPs).

At present, the dynamic description of municipal wastewater treatment processes, the change and transformation of wastewater components is mainly based on activated sludge models (ASMs)^5–7^. ASMs divide wastewater COD into biodegradable COD and non-biodegradable COD^8,9^. Biodegradable COD can be further divided into rapidly biodegradable COD (RBCOD) and slowly biodegradable COD (SBCOD). RBCOD is an easily available COD component that can be absorbed by heterotrophic microorganisms for biosynthesis and energy production, it directly determines the performance of nitrogen and phosphorus removal. SBCOD is divided into colloidal substrate and granular substrates, both of which are biodegradable substrates. These substances are often complex organic molecules and need to be decomposed by extracellular enzymes before they can be used by cells.

The design, control, and operation of a wastewater treatment system are largely dependent on how much is known about the characteristics of wastewater RBCOD and SBCOD components^10^. Therefore, it is particularly important to establish methods of wastewater characterization. Studies have shown that wastewater RBCOD and SBCOD components cannot be separated by physical–chemical methods^11^ Respirometric analysis method is a biological method that is widely used in wastewater COD component characterization. The respirometric method was confirmed as an effective method for determining RBCOD and SBCOD concentrations. The difference in biodegradation rate between wastewater RBCOD and SBCOD is reflected in the dynamic index of oxygen uptake rate (OUR)^12^. However, in the research and engineering practices associated with high-standard wastewater treatment, an increasing number of new phenomena and problems are becoming difficult to explain with conventional wastewater quality indicators, process theory and technical knowledge, among others, which restrict the operation process design, process optimization and regulation and stable operation of WWTPs^13^.

To meet the demand of high standard wastewater treatment and realize refined operational management, in this research, typical domestic wastewater is studied based on the respirometry method in order to establish a characterization method for wastewater COD components. The first goal of the present study was to analyse the influence of the initial substrate to biomass ratio (S_0_/X_0_) ratio on experimental OUR-t curves. Second, the respirometry method was utilized for the evaluation of RBCOD and SBCOD components. Finally, Mann–Kendall trend analysis method and a hydrolysis model were used to confirm the degradation time points of different components. Based on the results obtained in this study, a characterization analysis methodology for RBCOD and SBCOD components was proposed, which can effectively support operational process selection and process control technology research on WWTPs.

## Materials and methods

### Sampling of activated sludge and wastewater

Fresh activated sludge and raw wastewater were collected from the pilot A^2^/O system of the Research Center for Low Carbon Technology of Water Environment (Renmin University of China, Beijing). The characteristics of the experimental wastewater are listed in Table 1.

**Table 1:** Water characteristics of the experiment wastewater.

Constituents	Concentration	Mean
Chemical oxygen demand (mg/L)	283–524	418
Ammonia nitrogen (mg/L)	20.8–53.2	42.5
Total nitrogen (mg/L)	61.5–91.7	71.5
Total phosphorus (mg/L)	3.0–8.7	4.8
pH	6.53–7.55	7.31

In this study, 10 L activated sludge sample was taken from the aerobic terminal of the A^2^/O system, washed and concentrated, then the supernatant was removed. This operation was repeated three times. The obtained sample was put in a container and aerated for 3–4 h until the activated sludge exhibited endogenous respiration stage. Moreover, the mixed liquor volatile suspended solids (MLVSS) were adjusted to approximately 2000 mg/L.

### Respirometry experiments

**Figure 1:**
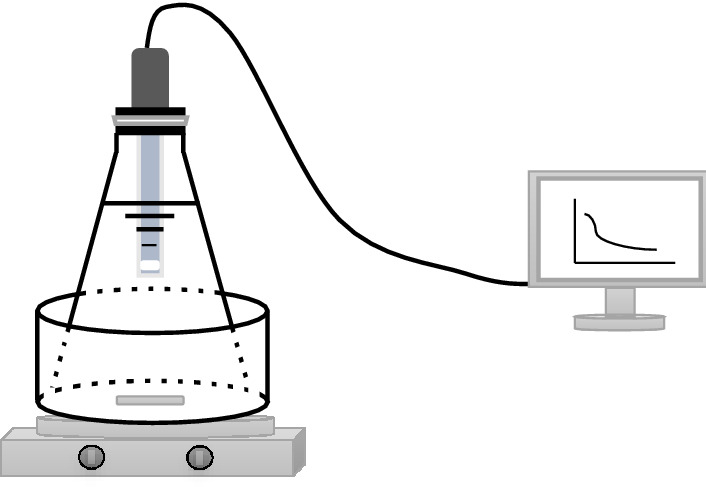
Static experiment device diagram.

Respiration rates were measured in a Plexiglas reactor with working volumes of 250 mL (Fig. 1). During the experiments, the temperature of the mixed liquid was kept constant at (20 ± 1) °C by placing the reactor in a constant-temperature water bath with magnetic stirring, and the pH was continuously controlled to 7.5 ± 0.3 by adding NaOH and HCl. The dissolved oxygen (DO) concentration was measured in the reactor by means of a WTW Multi3420 oxygen meter (Weilheim, Germany) and was continuously monitored with a paperless recorder^14^.

For all tests, allyl-thiourea (ATU) was added at a concentration of 20 mg/L to the mixed liquid to suppress nitrification activity^14^.

#### Determination of optimal S_0_/X_0_

Several respirometry experiments with different S_0_/X_0_ ratios were performed to analyse wastewater COD fractions: RBCOD and SBCOD^15,16^. In this study, according to the ratio of domestic wastewater (the equivalent weight of COD is approximately 400 mg/L) and MLVSS, S_0_/X_0_ was divided into five levels, namely, 0.2, 0.4, 0.6, 0.8 and 1.0 mgCOD/mgVSS, and then carried out batch experiments. First, the mixed liquid (200 mL) was added to the 250 mL Plexiglas reactor, with full aeration of the sludge mixture. Second, some ATU solution was added, inserted the DO probe of the WTW-type dissolved oxygen electrode into the Plexiglas reactor, sealed the bottle mouth, placed the bottle on the agitator, and stirred the mixture at a speed of 125 r/min to fully mix the sample. The running cycle was 4 min, the DO value was recorded every 10 s, and DO was continuously monitored with a paperless recorder. Third, the DO value from the good linear relationship of the DO-t curve was obtained, and the slope of the curve was estimated as OUR. An OUR-t curve was constructed and observed by the naked eye using step sample. The organic load of mixed liquid in RBCOD and SBCOD was particularly obvious, and the optimal S_0_/X_0_ could be determined.

#### Test method of COD component

Under the optimal S_0_/X_0_, the respirometry method was used for wastewater COD component characterization. The test procedure was as described in “Determination of optimal S_0_/X_0_” section, and the COD concentration corresponding to the test time was tested by sampling. During the oxygen uptake rate (OUR) test of the sludge mixture^17^, when nitrification is inhibited, the ideal OUR curve consists of three stages, namely, the rapidly biodegradable organic matter (RBCOD) degradation stage, slowly biodegradable organic matter (SBCOD) hydrolysis stage and activated sludge endogenous respiration stage, which can be obtained from the OUR-t experimental curve. In the process of OUR tests, 20 mL of mixed liquid was taken out and centrifuged every 5 min, and the supernatant was collected to determine the COD concentration in the wastewater. The time point (t_2_) when endogenous respiration levels were reached and the time point (t_1_) of the SBCOD degradation stage were determined and combined with the OUR-t curve. Equation fitting was then performed on the experimental curve and the concentrations of the RBCOD and SBCOD components were calculated by integrating the area of each region for the equation with a higher fitting degree.

### Chemical analyses

We analysed the COD concentration of the wastewater samples according to the standard method^18^. In this study, the organic matter in the wastewater was represented by the total chemical oxygen demand (TCOD), which could be divided into particulate COD (PCOD), colloidal COD (CCOD) and soluble COD (SCOD). The PCOD was the residual COD after filtration through a 1.5 μm filter, the SCOD was the COD that passed through a 0.45 μm filter, and the CCOD was calculated as the difference between TCOD and the sum of PCOD and SCOD^19,20^.

### Ethical standards

This study complied with all ethical standards in all phases of research.

## Results and discussion

### The optimal S_0_/X_0_

**Figure 2:**
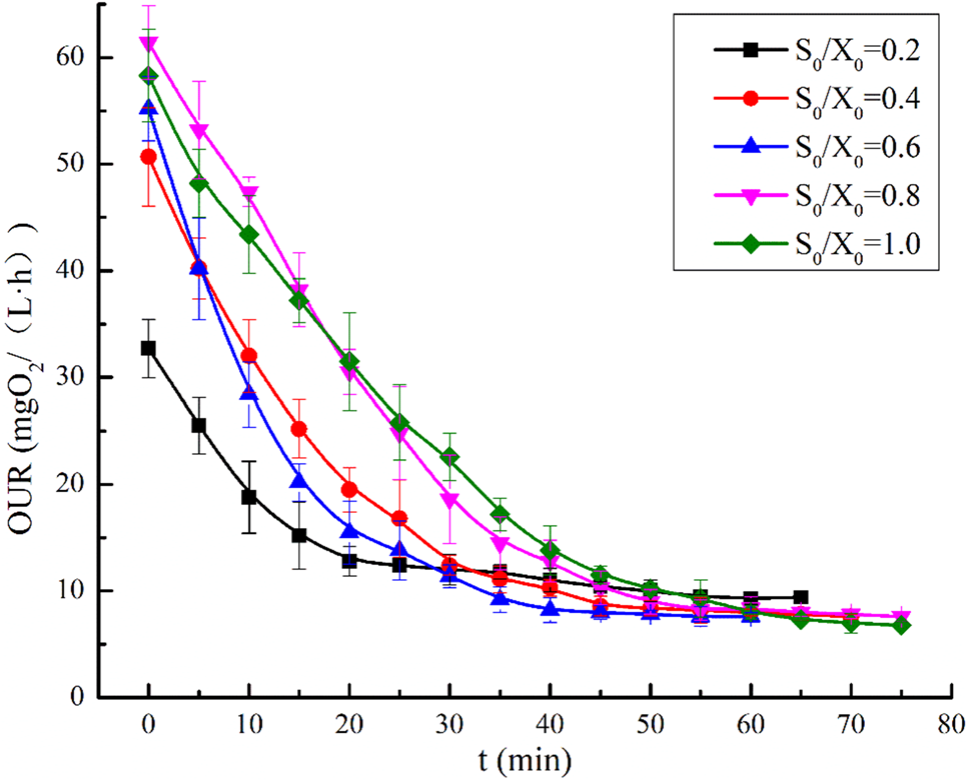
OUR curve of urban wastewater under different S_0_/X_0_.

Figure 2 illustrates the OUR-t curves of the respirometry experiments performed under different levels of S_0_/X_0,_ where the variation in the DO concentration data was determined under different operating conditions. As can be seen in Fig. 2, the OUR-t curves of wastewater COD degradation under different S_0_/X_0_ ratios showed the same change trend, but there were also obvious differences in segmented points and the resolvability of components. When S_0_/X_0_ was 0.2 mgCOD/mgVSS, from 0 to 55 min, the OUR decreased rapidly with reaction time from 32.5 to 7.5 mgO_2_/(L h). The segmentation was not obvious, which may be related to the low initial matrix concentration and the rapid completion of COD degradation reaction. The results showed that when the S_0_/X_0_ ratio was low, it was not conducive to the analysis of COD components. When the S_0_/X_0_ ratio was in the range of 0.8 to 1.0, the OUR decreased rapidly from 60 to 8 mgO_2_/(L h). After 40 min, the reaction exhibited the endogenous respiration level. From 0 to 40 min, the segmentation of the OUR-t curve was not obvious, so it was difficult to analyse the COD components. This may have been due to the high concentration of matrix and the interaction between the degradation and hydrolysis of different COD components. When S_0_/X_0_ ranged from 0.4 to 0.6, the OUR-t experimental curve was significantly segmented and could be clearly divided into three stages: the RBCOD degradation stage, SBCOD hydrolysis stage and endogenous respiration stage. The results showed that when the S_0_/X_0_ ratio was 0.4 to 0.6, the process of COD degradation could be clearly divided into three reaction stages by the respiration measurement method, and the segmentation was obvious, therefore, meeting the requirements for subsequent analysis of the COD components in wastewater influent.

Whether the OUR curve obtained is clearly segmented and has high resolvability is mainly determined by S_0_/X_0_. However, the optimal S_0_/X_0_ ratios obtained by applying respirometry experiments are not the same, and it is difficult to determine the optimal state^21^. When the S_0_/X_0_ ratio is higher, owing to the reaction time being sufficient, microorganisms have enough time to oxidize and degrade organic matter, resulting in changes in the maximum specific growth rate of the microorganisms and the degradation rate of the substrate, and further leading to the rapid growth of microorganism via mass multiplication^22^. Moreover, the slower growth of microorganisms is inhibited, which results in changes in microbial community structure in the activated sludge. When the S_0_/X_0_ ratio is lower, the reaction is completed quickly, and it is difficult to observe the rapid oxidation process of wastewater RBCOD components. Hence, the OUR value of the process of microbial degradation is close to the endogenous respiration^23^, and the component' information cannot be obtained. Some studies have shown that the S_0_/X_0_ ratio can be 0.6 mgCOD/mgVSS. However, other studies showed that when the S_0_/X_0_ ratio is maintained at 0.45–1.0 mgCOD/mgVSS^24^, a clear OUR curve can be obtained and the deviation maintained at 5–10%. A reasonable S_0_/X_0_ ratio is related to the source and nature of the experimental wastewater and sludge. Therefore, it is very important to determine the optimal S_0_/X_0_ ratio through experiments.

### Measurement of COD components

**Figure 3:**
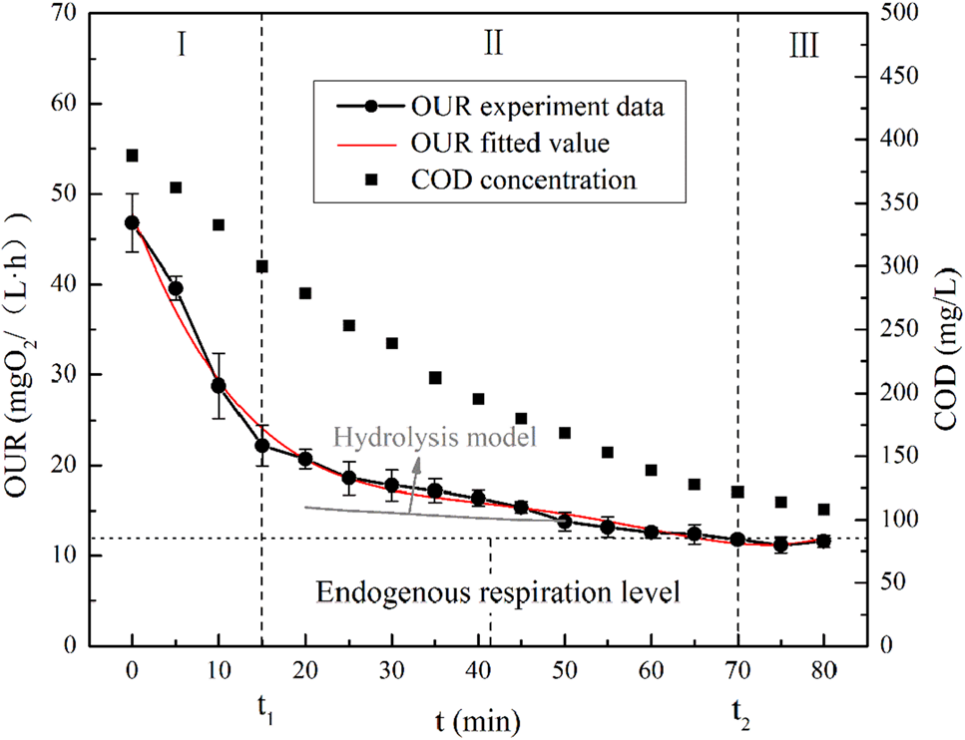
Complete OUR curve of wastewater COD degradation.

According to the optimal S_0_/X_0_ determination experiment, we confirmed that the optimal S_0_/X_0_ ratio was 0.5 mgCOD/mgVSS, and the OUR-t curve of wastewater COD degradation was shown in Fig. 2. The complete OUR-t curve of COD degradation showed a decreasing trend with reaction time. It was evidently composed of two external respiration (OUR_ex_) stages and an endogenous respiration (OUR_en_) stage based on the significant difference in respiration rates. The first stage was the utilization of rapidly biodegradable matrix by microorganisms. Due to the sufficient matrix in this stage, RBCOD and DO were consumed rapidly by microorganisms, and the OUR was at a peak at this time. With the gradual decrease in RBCOD, the OUR decreased continuously. As shown in Fig. 3, in this stage, the OUR decreased rapidly from 46.8 to 22.2 mgO_2_/(L h). After 15 min of reaction, owing to the RBCOD reaction generally being complete, the system begins to enter the SBCOD degradation stage. Because the hydrolysis reaction is a slow process, the OUR decreased slowly. With the hydrolysis and oxidation of SBCOD, OUR decreased rapidly from 22.2 to 11.8 mgO_2_/(L h), and the reaction time of the process was approximately 5–7 times longer than the previous stage, approximately 70 min. During this reaction stage, SBCOD is hydrolysed to RBCOD with small molecules, and the OUR response is caused by the degradation of RBCOD. Because the hydrolysis process is relatively slow, the OUR-t curve shows a slow downward trend. After 70 min, the substrate in the wastewater almost completely reacts and begins to enter the endogenous respiration stage. At this time, microorganisms in the activated sludge use only their own stored substances for respiration, and the OUR remained at approximately 11.5 mgO_2_/(L h), which was close to the level of endogenous respiration measured in the early stage of the experiment. The biological or respirometric component of the characterization method was based on the measurement of the biomass response during substrate degradation in either continuous flow or batch conditions. Based on the respirometry setup, the wastewater RBCOD and SBCOD are easy to measure. During the first stage, this allows the microbial population to consume RBCOD faster and to be detected by the OUR measurement. The profile of fitting can be used to calculate the concentration of RBCOD and SBCOD components. In this experiment, the OUR fitting profile can be described as: y = 8.72 x^4^ − 0.001 x^3^ + 0.077 x^2^ −  2.52 x + 47.81, R^2^ = 0.98817.

**Figure 4:**
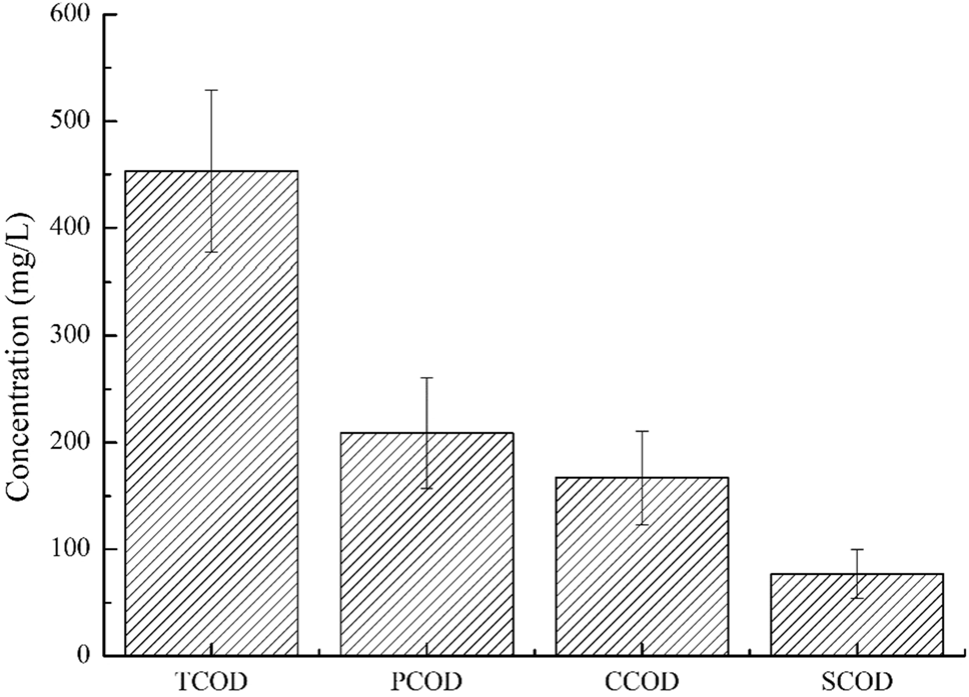
PCOD, CCOD and SCOD components concentration of wastewater samples.

According to the segmentation of the OUR-t curve, the degradation of COD in each stage was analysed. The concentration of COD in the mixture decreased from 387.5 to 108.2 mg/L during the endogenous respiration stage. From 0 to 15 min, the concentration of COD rapidly decreased from 387.5 to 299.6 mg/L, and the degradation rate was approximately 5.86 mg/(L min). From 15 to 70 min, the COD concentration decreased from 299.6 to 122.1 mg/L, and the COD degradation rate was approximately 3.22 mg/(L min). The COD degradation rate was approximately 1.39 mg/(L min) in the 70 to 80 min interval. The results showed that under the optimal S_0_/X_0_, the OUR-t curve could be divided into three stages, which may represent different COD components. Similarly, we analysed the PCOD, CCOD and SCOD of wastewater influent. As expected, TCOD is generally divided into three fractions, namely, PCOD, CCOD and SCOD. Moreover, the concentration of three types of COD has a strong correlation with wastewater RBCOD and SBCOD. The wastewater PCOD, CCOD and SCOD fractions were 46.14 ± 11.48%, 36.87 ± 9.71%, and 16.70 ± 5.08%, respectively (Fig. 4).

RBCOD consists of smaller molecules, which are mostly volatile fatty acids (VFAs) and low molecular weight carbohydrates, and is considered soluble organic matter with a particle size less than 0.45 µm. Reports showed that the percentage of the suspended fraction in the TCOD of raw domestic wastewater was 65–79%, while the dissolved fraction was 21–35%^8^. Biodegradable organic components represent an average of 39% of all organic matter in wastewater, and non-biodegradable components, approximately 61%^25^. This result is basically in accord with that for the three types of COD.

### Methods of characterizing wastewater COD components

In the process of biotransformation, SBCOD needs to be hydrolysed into RBCOD with small molecule and easy biodegradation, before it can be absorbed and utilized by microorganism in activated sludge, finally, realizing the degradation of pollutants. The results showed that the degradation rates of RBCOD and SBCOD differed by 1–2 orders of magnitude, so the two parts could be effectively distinguished theoretically. A complete OUR-t curve of wastewater COD degradation theoretically includes four stages: the RBCOD degradation stage, nitrification stage, SBCOD degradation stage and endogenous respiration stage. The biological inhibitor ATU (20 mg/L) can inhibit nitrification. When nitrification is inhibited, the OUR-t curve can be divided into three stages. Among them, the degradation regulation of RBCOD accords with the Monod equation, and that of SBCOD accords with the hydrolysis rate equation. Therefore, only the time point (t_1_) of the SBCOD degradation stage and the time point (t_2_) when endogenous respiration levels were reached can be determined, and RBCOD and SBCOD can be solved quantitatively by an integral equation.

#### Determination of endogenous respiration levels (the time point (t_1_))

In this study, after the comparison and selection of the analysis method, the non-parametric Mann–Kendall test^26–28,29^ was used to determine the time point (t_2_) when the endogenous respiration level was reached. The Mann–Kendall test is used to determine whether a time series has a monotonic upward or downward trend. It does not require that the data be normally distributed or linear, and it is convenient and quick.

In the Mann–Kendall test, the time series {x_1_, x_2_, …, x_n_} represents n individual, independent and uniformly distributed samples of random variables, which are recorded as the null hypothesis H_0_. The alternative hypothesis H_1_ is a trend in the two-sided test or an upward (or downward) trend in the one-sided test. For all values of i, when j ≤ n and i ≠ j, the distributions of x_i_ and x_j_ are not the same. The trend test statistic S is calculated as follows:

1 $$S = \sum\limits_{i = 1}^{{{\text{n - }}1}} {\sum\limits_{j = i + 1}^{{\text{n}}} {Sgn\,\left( {X_{{\text{j}}} - X_{{\text{i}}} } \right)} }$$

where Sgn is a symbolic function

2 $${\text{Sgn}}\left( {X_{{\text{j}}} - X_{{\text{i}}} } \right) = \left\{ {\begin{array}{*{20}l} 1 \hfill & {\left( {X_{{\text{j}}} - X_{{\text{i}}} } \right) > 0} \hfill \\ 0 \hfill & {\left( {X_{{\text{j}}} - X_{{\text{i}}} } \right) = 0} \hfill \\ { - 1} \hfill & {\left( {X_{{\text{j}}} - X_{{\text{i}}} } \right) < 0} \hfill \\ \end{array} } \right.$$

where S is a normal distribution, with a mean value of zero and a variance Var(S) = n(n − 1)(2n + 5)/18. When n > 10, the statistic Z_MK_ converges to the standard normal distribution, the null hypothesis is that the sequence has no trend, and the bilateral trend test method is used.

3 $$Z_{MK} = \left\{ {\begin{array}{*{20}l} {\frac{S - 1}{{\sqrt {V{\text{ar}}\left( S \right)} }}} \hfill & {S > 0} \hfill \\ 0 \hfill & {S = 0} \hfill \\ {\frac{S + 1}{{\sqrt {V{\text{ar}}\left( S \right)} }}} \hfill & {S < 0} \hfill \\ \end{array} } \right..$$

In the bilateral trend test, at a given α confidence level, when {|Z_MK_|} ≥ Z_1 − α/2_, the null hypothesis is rejected; that is, at the α confidence level, there is a significant increase or decrease in the OUR series data trend. If Z_MK_ > 0, there is an upward trend, while if Z_MK_ < 0, there is a downward trend. When {|Z_MK_|} < Z_1 − α/2_, the null hypothesis that the change trend is not significantly accepted.

In this study, the Mann–Kendall test, the mutation test method, and Excel statistical software were used in combination. The Mann–Kendal test or mutation test was carried out from the end of the obtained OUR curve to the beginning. The portion of the respiratory rate curve without an obvious change trend was the endogenous respiration phase, reflecting the time point (t_2_) when the endogenous respiration level was reached. The analysis of the Mann–Kendal test results is shown in Table 2.

**Table 2:** Analysis of trend testing in Excel.

Time/min	Time	0	5	10	15	20	25	30	35	40	45
	Mean mg/(L h)	46.8	39.6	28.8	22.2	20.7	18.6	17.8	17.2	16.4	15.4
0	46.8		− 7.2	− 18	− 24.6	− 26.1	− 28.2	− 29	− 29.6	− 30.4	− 31.4
5	39.6			− 10.8	− 17.4	− 18.9	− 21	− 21.8	− 22.4	− 23.2	− 24.2
10	28.8				− 6.6	− 8.1	− 10.2	− 11	− 11.6	− 12.4	− 13.4
15	22.2					− 1.5	− 3.6	− 4.4	− 5	− 5.8	− 6.8
20	20.7						− 2.1	− 2.9	− 3.5	− 4.3	− 5.3
25	18.6							− 0.8	− 1.4	− 2.2	− 3.2
30	17.8								− 0.6	− 1.4	− 2.4
35	17.2									− 0.8	− 1.8
40	16.4										− 1
45	15.4										
50	13.8										
55	13.2										
60	12.6										
65	12.4										
70	11										
75	11.2										
80	11.3										

Time/min	Time	50	55	60	65	70	75	80	BD (+)	BE (−)	
	Mean mg/(L h)	13.8	13.2	12.6	12.4	11	11.2	11.3			
0	46.8	− 33	− 33.6	− 34.2	− 34.4	− 35.8	− 35.6	− 35.5	0	16	
5	39.6	− 25.8	− 26.4	− 27	− 27.2	− 28.6	− 28.4	− 28.3	0	15	
10	28.8	− 15	− 15.6	− 16.2	− 16.4	− 17.8	− 17.6	− 17.5	0	14	
15	22.2	− 8.4	− 9	− 9.6	− 9.8	− 11.2	− 11	− 10.9	0	13	
20	20.7	− 6.9	− 7.5	− 8.1	− 8.3	− 9.7	− 9.5	− 9.4	0	12	
25	18.6	− 4.8	− 5.4	− 6	− 6.2	− 7.6	− 7.4	− 7.3	0	11	
30	17.8	− 4	− 4.6	− 5.2	− 5.4	− 6.8	− 6.6	− 6.5	0	10	
35	17.2	− 3.4	− 4	− 4.6	− 4.8	− 6.2	− 6	− 5.9	0	9	
40	16.4	− 2.6	− 3.2	− 3.8	− 4	− 5.4	− 5.2	− 5.1	0	8	
45	15.4	− 1.6	− 2.2	− 2.8	− 3	− 4.4	− 4.2	− 4.1	0	7	
50	13.8		− 0.6	− 1.2	− 1.4	− 2.8	− 2.6	− 2.5	0	6	
55	13.2			− 0.6	− 0.8	− 2.2	− 2	− 1.9	0	5	
60	12.6				− 0.2	− 1.6	− 1.4	− 1.3	0	4	
65	12.4					− 1.4	− 1.2	− 1.1	0	3	
70	11						0.2	0.3	2	0	
75	11.2							0.1	1	0	
80	11.3							SUM	3	133	

Note: (1) Z is a positive value that indicates an upward trend, and a negative value indicates a downward trend. (2) When the absolute value of Z is greater than or equal to 1.28, 1.64, and 2.32, it passes the significance test at 90%, 95%, and 99% reliability respectively.

After the calculations, the parameter n is 17, S is − 130, Var(S) is 589, and Z is − 5.3. The results show that |Z|> 2.32 > 0, which passed the significance test at 99% reliability. Since Z = −5.32, the OUR exhibits an upward trend.

In the non-parametric Mann–Kendall test, we set the time series as {x_1_, x_2_, …, x_n_}; S_k_ represents the cumulative number of the ith sample x_i_ > x_j_ (1 ≤ j ≤ i), and defines the statistic:

4 $$S_{{\text{k}}} = \sum\limits_{{{\text{i}} = {1}}}^{{\text{k}}} {{\text{r}}_{{\text{i}}} } \;\;\;\;\;\;\;\;{\text{r}}_{{\text{i}}} = \left\{ \begin{array}{ll} 1,&\quad x_{i} > x_{j} \hfill \\ 0,&\quad x_{i} \le x_{j} \;\;\; \hfill \\ \end{array} \right.\left( {{\text{j}} = 1,2\ldots,i;k = 1,2,\ldots,n} \right).$$

Under the assumption of random independence of the time series, the mean and variance of S_k_ are: E[S_k_] = k(k − 1/4), var[S_k_] = k(k − 1)(2 k + 5)/72, Among them, 1 ≤ k ≤ n.

After standardizing S_k_, we obtain:

5 $$UF_{k} = \frac{{\left( {S_{k} - E[S_{k} ]} \right)}}{{\sqrt {{\text{var}} [S_{k} ]} }}.$$

The Mann–Kendall test is performed from the end of the obtained OUR-t curve to the beginning, and the point of intersection within the confidence interval was determined as the mutation point t_2_. The mutation test results from Excel are shown in Tables 3 and 4.

**Table 3:** Sequential column data of OUR.

Time/min	Mean /mgO_2_/(L h)	k	r	S_k_	E (S_k_)	Var (S_k_)	UF (S_k_)
0	46.8	1	0	0	0	0.00	0.00
5	39.6	2	0	0	0.5	0.25	− 1.00
10	28.8	3	0	0	1.5	0.92	− 1.57
15	22.2	4	0	0	3	2.17	− 2.04
20	21.7	5	0	0	5	4.17	− 2.45
25	18.6	6	0	0	7.5	7.08	− 2.82
30	17.8	7	0	0	10.5	11.08	− 3.15
35	17.9	8	1	1	14	16.33	− 3.22
40	16.4	9	0	1	18	23.00	− 3.54
45	15.4	10	0	1	22.5	31.25	− 3.85
50	13.8	11	0	1	27.5	41.25	− 4.13
55	13.2	12	0	1	33	53.17	− 4.39
60	12.6	13	0	1	39	67.17	− 4.64
65	12.4	14	0	1	45.5	83.42	− 4.87
70	11	15	0	1	52.5	102.08	− 5.10
75	11.2	16	1	2	60	123.33	− 5.22
80	11.3	17	2	4	68	147.33	− 5.27

**Table 4:** Inverse sequence computation data of OUR.

Time/min	Mean/mgO_2_/(L h)	k′	r′	S_k_′	E(S_k_)′	Var(S_k_)′	UF(S_k_)′
0	11.3	1	0	0	0	0	0
5	11.2	2	0	2	0.5	0.25	3.00
10	11	3	0	3	1.5	0.92	1.57
15	12.4	4	3	4	3	2.17	0.68
20	12.6	5	4	8	5	4.17	1.47
25	13.2	6	5	10	7.5	7.08	0.94
30	13.8	7	6	12	10.5	11.08	0.45
35	15.4	8	7	14	14	16.33	0.00
40	16.4	9	8	16	18	23.00	− 0.42
45	17.9	10	9	18	22.5	31.25	− 0.80
50	17.8	11	9	20	27.5	41.25	− 1.17
55	18.6	12	11	21	33	53.17	− 1.65
60	20.7	13	12	24	39	67.17	− 1.83
65	22.2	14	13	26	45.5	83.42	− 2.14
70	28.8	15	14	28	52.5	102.08	− 2.42
75	39.6	16	15	30	60	123.33	− 2.70
80	46.8	17	16	32	68	147.33	− 2.97

#### Determination of SBCOD hydrolysis stage (the time point (t_1_))

Studies have shown that the degradation of SBCOD conforms to the hydrolysis rate equation (Eq. 6). Hydrolysis refers to the process by which SBCOD decomposes into small-molecular organics that can be used by microorganisms in activated sludge^24,30–32^.

The hydrolysis rate equation can be expressed as:

6 $$\frac{d{X}_{s}}{dt}=-{K}_{h}\frac{{X}_{s}(t)/{X}_{H}}{{X}_{s}(t)/{X}_{H}+{K}_{h}}\times {X}_{H}.$$

The quantitative relationship between the OUR and matrix is:

7 $$OUR\left(t\right)=-\frac{d{S}_{s}}{dt}(1-{Y}_{H}).$$

Combining the above two equations, we obtain Eq. (8):

8 $$OUR\left(t\right)=\frac{{K}_{h}\times {X}_{H}(1-{Y}_{H})\times {X}_{s}(t)}{{X}_{s}(t)/{X}_{H}+{K}_{h}}$$

where K_h_ is the maximum specific hydrolysis rate, gCOD_Xs_/(gCOD_XH_·d); X_H_ is the concentration of heterotrophic bacteria, mgCOD/L; and Y_H_ is the yield coefficient of heterotrophic bacteria, g/g.

To determine the time point (t_1_) when the SBCOD degradation stage starts, from the time point (t_2_) onwards, a segment of the OUR sequence is taken, equation fitting of the data is performed, and whether to continue taking OUR data forward is determined according to the fitting error. When the OUR value no longer conforms to the fitting equation, the OUR no longer conforms to the law of hydrolysis, indicating fitting distortion, and the time point when fitting distortion starts is considered the time point (t_2_).

#### Quantitative calculation of RBCOD and SBCOD concentrations

The quantitative relationship between the OUR and matrix is shown in Eq. (9), which can be used to calculate the initial concentrations of RBCOD and SBCOD components.

9 $$\mathrm{S}=\frac{\int _{{\mathrm{t}}_{\mathrm{initial}}}^{{\mathrm{t}}_{\mathrm{final}}}\mathrm{OURdt}}{1-{\mathrm{Y}}_{\mathrm{H}}}$$

where S is the initial concentration of the substrate, mgCOD/L, and Y_H_ is the yield coefficient of heterotrophic bacteria, 0.68 in this study.

The selection of suitable methods for characterizing wastewater components plays a key role in the design, control, and operation of WWTPs^10^. In this study, based on the above analysis, the characteristic analysis steps of wastewater COD components are summarized as follows:

Step 1: The optimal S_0_/X_0_ was confirmed. Under this ratio, we determined the OUR-t curve of the wastewater influent COD component and used the Mann–Kendall test to determine the time point (t_2_) when the endogenous respiration stage began.

Step 2: The degradation regulation of SBCOD conforms to the hydrolysis model. The hydrolysis model is used for fitting, and the measured data are verified to determine the time point (t_1_) of the SBCOD degradation stage.

Step 3: After determining the time point (t_2_) when endogenous respiration levels are reached and the time point (t_1_) of the SBCOD degradation stage, OUR-t curve fitting is performed, followed by determination of the quantitative relationship between the OUR and matrix degradation to calculate the RBCOD and SBCOD component concentrations.

## Conclusions

In this study, the conclusions are as follows:

1. The accuracy of respirometric evaluation of wastewater RBCOD and SBCOD is strongly related to the experimental conditions and S_0_/X_0_ ratio. In this work, a satisfactory evaluation of wastewater components was performed with a S_0_/X_0_ ratio of approximately 0.5 mgCOD/mgVSS.
2. Using the Mann Kendall test and Hydrolysis model, the time point (t_2_) when endogenous respiration levels are reached and the time point (t_1_) of the SBCOD degradation stage were determined. The wastewater RBCOD and SBCOD concentrations were 37.13 and 144.38 mg/L, respectively. The result is basically in accord with that for the three types of COD, and shows the effectiveness of the analytical methods.
3. The analysis steps of wastewater characterization methods are summarized as follows: determine the OUR-t curve under the optimal S_0_/X_0_ ratio, use the Mann–Kendall test to determine the time point (t_2_) of the endogenous respiration phase, and use the hydrolysis model to determine the time point (t_1_) of the SBCOD degradation stage; then, evaluate the quantitative relationship between the OUR and matrix degradation, and calculate the RBCOD and SBCOD concentrations.
